# Utility of normalized genome quantification of *Helicobacter pylori* in gastric mucosa using an in-house real-time polymerase chain reaction

**DOI:** 10.1371/journal.pone.0178674

**Published:** 2017-06-02

**Authors:** Ana Morilla, Santiago Melón, Marta E. Álvarez-Argüelles, Edisa Armesto, Henar Villar, María de Oña

**Affiliations:** 1 Department of Microbiology, Hospital Universitario San Agustín, Avilés, Spain; 2 Department of Microbiology, Hospital Universitario Central de Asturias, Oviedo, Spain; 3 Department of Gastroenterology, Hospital Universitario San Agustín, Avilés, Spain; Kliniken der Stadt Köln gGmbH, GERMANY

## Abstract

Traditional diagnostic assays for *Helicobacter pylori* detection have their limitations. Molecular methods can improve both diagnosis and understanding of gastric diseases. Here we describe an in-house quantitative real-time polymerase chain reaction (q-rt-PCR) for the detection of *H*. *pylori* in gastric biopsies which has been developed and has a detection limit of 10 copies, the specificity of which was tested against other gastric colonizer bacteria. In this study, 199 gastric biopsies from adults with different clinical gastric symptoms were examined. Biopsies were obtained during endoscopy and the following tests performed: rapid urease testing (RUT), culture and q-rt-PCR. *H*. *pylori* bacterial load expressed as bacterial load per 10^5^ cells was calculated using a standard curve. *H*. *pylori* was isolated in 41% of patients, RUT was positive in 32% and bacterial genome was detected in 45% (p = 0.010). Concordance between traditional invasive microbiological methods used together and q-rt-PCR was almost 100%. Bacterial load in patients with positive RUT was significantly higher than those where it was negative (p<0.0001). There were also significant differences between bacterial load in patients with more than one positive assay versus those where only one method was positive (p = 0.006). The in-house q-PCR developed here is quick and inexpensive, and allows accurate diagnosis of *H*. *pylori* infection. It also permits normalized bacterial load quantification, which is important to differentiate between asymptomatic colonisation and infection.

## Introduction

*Helicobacter pylori* (*H*. *pylori*) is one of the most prevalent pathogens worldwide, infecting an estimated 50% of the global population [1,2]. It produces diseases of the upper gastrointestinal tract such as chronic active gastritis, peptic ulcer disease (duodenal and gastric), mucosa-associated lymphoid tissue (MALT) and extra-digestive diseases [3,4]. It is also considered a human carcinogen [5].

*H*. *pylori* is a motile, spiral microaerobic Gram-negative bacillus which adheres to the gastric mucosa of the epithelial layer of the stomach. It produces urease enzyme, which is important for colonisation, in abundance, and this can be used to detect the organism.

There are many testing methods available for the detection of *H*. *pylori*, both invasive and non-invasive. Invasive methods, which use an endoscope, include rapid urease testing (RUT), culture and histology. Non-endoscopic approaches include faecal antigen detection, serologic testing and urea breath testing [6,7]. Which of these tests to use depends on cost and availability, as well as on the prevalence of the *H*. *pylori* infection [1,6]. Furthermore, none of these techniques accurately quantifies the *H*. *pylori* present in test samples, information which could be important for the clinical management of the infection. Molecular methods for detection of *H*. *pylori* may constitute a more reliable approach [8,9]. Nowadays there are many commercial molecular tests available with high sensitivity and specificity, but they are costly, which puts them beyond the reach of some clinical laboratories.

The aim of this study was to develop a proprietary quantitative real time PCR, analyse its utility and compare it with culture and RUT methods for the detection of *H*. *pylori* in gastric biopsies.

## Materials and methods

### 2.1. Patients

This study comprised samples from 199 symptomatic patients attending the Gastroenterology Unit of the Hospital Universitario San Agustín (Avilés, Spain). Written informed consent was obtained from each patient, and the protocol was approved by the Research Ethics Committee of the Hospital. At the time that samples were collected, patients had not received proton-pump inhibitor drugs (PPIs) or antibiotics for at least 2 weeks. Clinical features of the patients recruited are presented in Table 1.

**Table 1:** Demographic and clinical characteristics of patients.

	Male (N = 75)	Female (N = 124)	Total (N = 199)
Age (mean ± SD (range))	56.1±16.3 (14–87)	57.6±18.6 (15–92)	57.0±17.7 (14–92)
**Result of endoscopy**			
Normal/Gastritis	30 (40.0%)	72 (58.1%)	102 (51.3%)
Gastric ulcer	9 (12.0%)	23 (18.5%)	23 (16.1%)
Duodenal ulcer	29 (38.7%)	27 (21.8%)	56 (28.1%)
Healed ulcer	7 (9.3%)	2 (1.6%)	9 (4.5%)

All the data were anonymised.

### 2.2. Gastric biopsy specimens and classical diagnosis methods

All the samples were taken from stomach antrum. All samples were transported to the Microbiology Laboratory in *Portagerm pylori* transport medium (*BioMérieux*, France). Biopsies were processed as follows: single fragments were cultured in both nonselective and selective media (*Columbia agar* with 5% sheep blood and *Pylori agar*, respectively, both from B*ioMérieux*, France) and then incubated for 10 days at 37°C in a microareophilic atmosphere (5% O_2_, 10% CO_2_ and 85% N_2_). Isolates were identified as *H*. *pylori* based on colony morphology, positive biochemical reactions for urease, catalase and oxidase tests, and characteristic Gram staining. Another biopsy fragment was used for rapid urease test (RUT) which was inoculated into urea broth and incubated for 2 days at 37°C in an aerophilic atmosphere. The test result was considered positive if there was a colour change from yellow-gold to pink-red, which is due to an increase in pH induced by urease enzyme production. Finally, another fragment was stored at -20°C until DNA extraction. Next, PCR was performed as described below. Where only one single piece of gastric biopsy was received, these were divided in the laboratory using a sterile scalpel.

### 2.3. Q-PCR methods

#### (i) DNA extraction

DNA was extracted from the biopsies after an overnight enzymatic digestion with 0.25% trypsin at 37°C. A proteinase K method was used: 200μL of lysis solution (10mM Tris-HCl pH 8.3, 50mM potassium chloride, 2.5mM magnesium chloride, 0.5% Igepal, 0.5% Tween 20 and 10μg of proteinase K) was added to each sample, mixed and incubated for 40 minutes at 60°C, then 10 minutes at 96°C to inactivate proteinase K. Samples were then stored at -20°C until use.

#### (ii) Quantification of microorganism

An in-house system was designed to amplify a fragment of the *ureA* gene of *H*. *pylori* using the software package Primer Express 3.0.1 (*Applied Biosystems*, California). The primers and TaqMan FAM-labelled probe used are shown in Table 2. The quantitative PCR reaction was carried out in a total volume of 11μL: 5μL of Brilliant III Ultra fast QPCR Master Mix (*Agilent Technologies*, USA), 1μL of 900nM primers and 250nM probe, and 5μL of purified DNA sample. The reaction was run on a Cobas 480 real-time PCR platform (*Roche Diagnostics GmbH*, Germany). PCR amplification comprised an initial denaturation cycle at 95°C for 10 minutes, followed by 40 amplification cycles consisting of 95°C for 10 seconds, annealing at 55°C for 22s and extension at 60°C for 30s.

**Table 2:** Nucleotide sequences of primers and probes.

	Primers and Probes	Sequences
UreA *H*. *pylori*	Forward target DNA primer	5'-TGCAAGAAGGGCGCACTCT-3'
	Reverse target DNA primer	5'-CCATCAGGAAACATCGCTTCA-3'
	Target DNA probe	(FAM)5’-CCGGATGATGTGATGGA-3’
Beta	Forward target DNA primer	5'-ACACAACTGTGTTCACTAGC-3'
	Reverse target DNA primer	5'-CCAACTTCATCCACGTTCACC-3'
	Target DNA probe	(FAM)5’-TGCATCTGACTCCTGAGGA-3’

A standard curve was performed using serial dilutions from 10 to 10^10^ of amplicon-based positive controls in order to determine the sensitivity of the assay.

The specificity was evaluated using bacterial strains of various microorganisms frequently found in the human gastrointestinal microbiome: *Pseudomonas aeruginosa*, *Proteus mirabilis*, *Escherichia coli*, *Campylobacter jejuni*, *Enterococcus faecalis*, *Staphylococcus spp*., *Citrobacter spp*. and *Morganella morganii*.

In addition, the human ß-globine gene was quantified in each sample in order to evaluate sample cellularity and correct for the different sizes of gastric biopsies in order to normalise bacterial load (Table 2).

Bacterial count as copies/10^5^cells and log was calculated using the quotient between the standard curve of the controls and the curve for the β-globine gene described in Álvarez-Argüelles et al.[10].

### 2.4. Analysis of results

A patient was considered to be infected with *H*. *pylori* when at least one of the assays was positive.

Statistical tests were performed using *SPSS software* (version 23.0, *SPSS*, Chicago, IL, USA). Agreement between diagnostic tests was assessed by *Cohen’s kappa coefficient*. *H*. *pylori* status in relation to gastroduodenal diseases was assessed by *Chi square test*. Average copy number was compared with the *Mann-Whitney rank sum* test and *Kruskal-Wallis H test* for two, or more variables, respectively. All p-values were two sided and considered significant when below 0.05.

## Results

### 3.1. Sensitivity and specificity of PCR

A ten-fold dilution series of *H*. *pylori* standard DNA was tested by the q-PCR assay to evaluate the sensitivity of the system, and the detection limit was found to be 10 copies.

**Fig 1:**
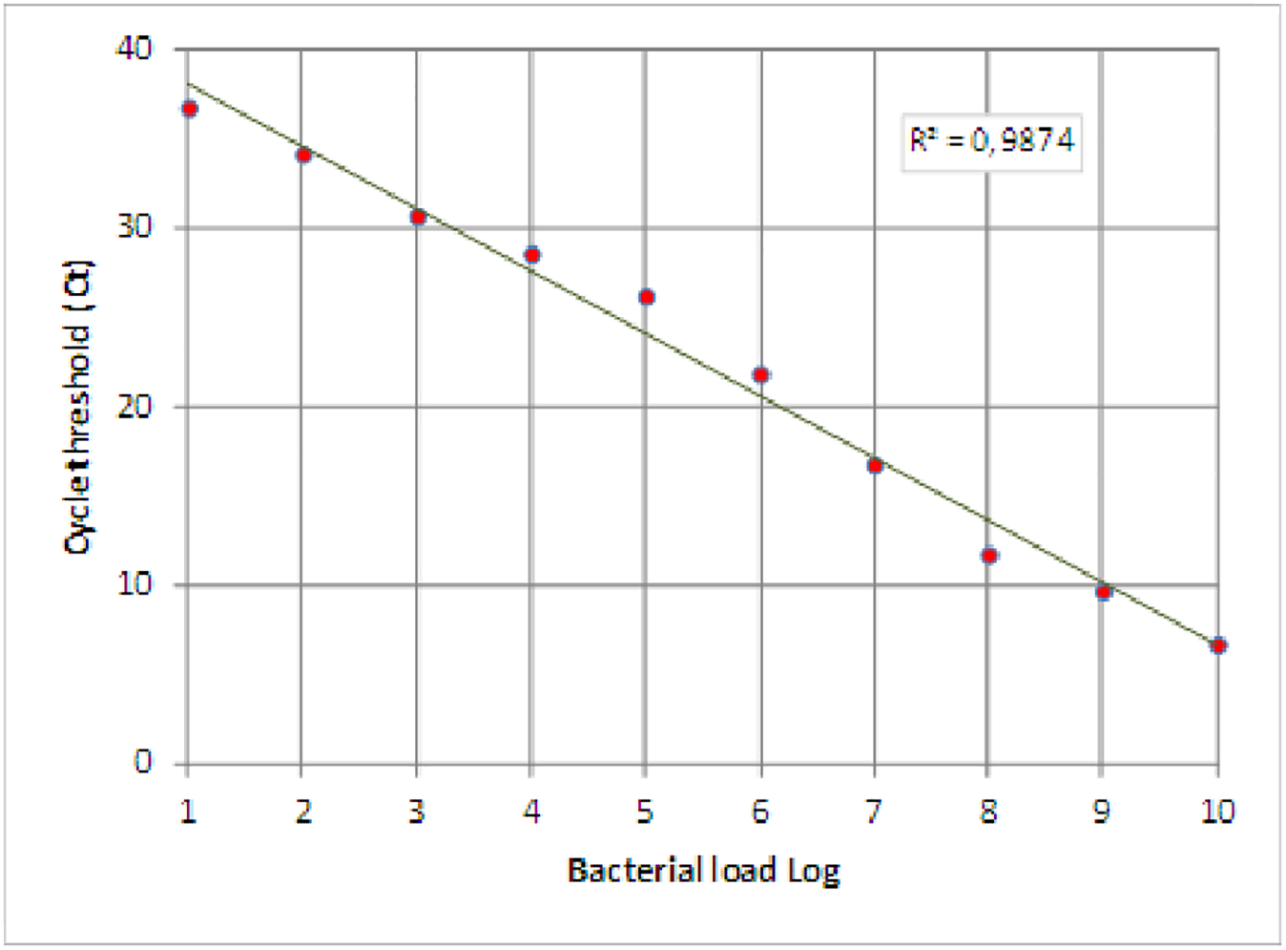
Standard curve of H. pylori q-PCR assay.

By plotting the log DNA copies count against threshold cycle (Ct) values, a standard curve was performed (Fig 1).

Specificity of q-PCR was proven using DNA from several other bacteria, none of which gave any amplification signal.

Amplification of human ß-globine gene was positive in all the biopsies, indicating successful DNA extraction.

### 3.2. Sample results

*H*. *pylori* infection was diagnosed by one or more of the three methods in 91 (46%) of the total 199 patients. It was found in 43 (42%) patients with gastritis/normal endoscopic findings, 15 (47%) patients with gastric ulcer, 31 (55%) patients with duodenal ulcer and 2 (22%) patients with healed ulcer (p = ns).

In terms of the diagnostic method used and endoscopy results, Table 3 shows that *H*.*pylori* was detected in a total of 90 samples (45%) by PCR, 81 (41%) by culture and 64 (32%) by RUT. Using traditional methods (culture plus RUT*) H*. *pylori* was detected in a total of 89 (45%) patients. RUT alone showed statistically significant lower sensitivity than either q-PCR or culture alone (p = 0.010).

**Table 3:** *H*. *pylori* results by different diagnostic test according to result of endoscopy.

Endoscopic findings	Normal/Gastritis (n = 102)	Gastric ulcer (n = 32)	Duodenal ulcer (n = 56)	Healed ulcer (n = 9)	TOTAL (n = 199)
q-PCR	42 (41%)	15 (47%)	31 (55%)	2 (22%)	90 (45%)
Culture	37 (36%)	14 (44%)	28 (50%)	2 (22%)	81 (41%)
RUT	30 (29%)	10 (31%)	22 (39%)	2 (22%)	64 (32%)^a^
Culture+RUT	41 (40%)	15 (47%)	31 (55%)	2 (22%)	89 (45%)

^**a**^p = 0,010

**Fig 2:**
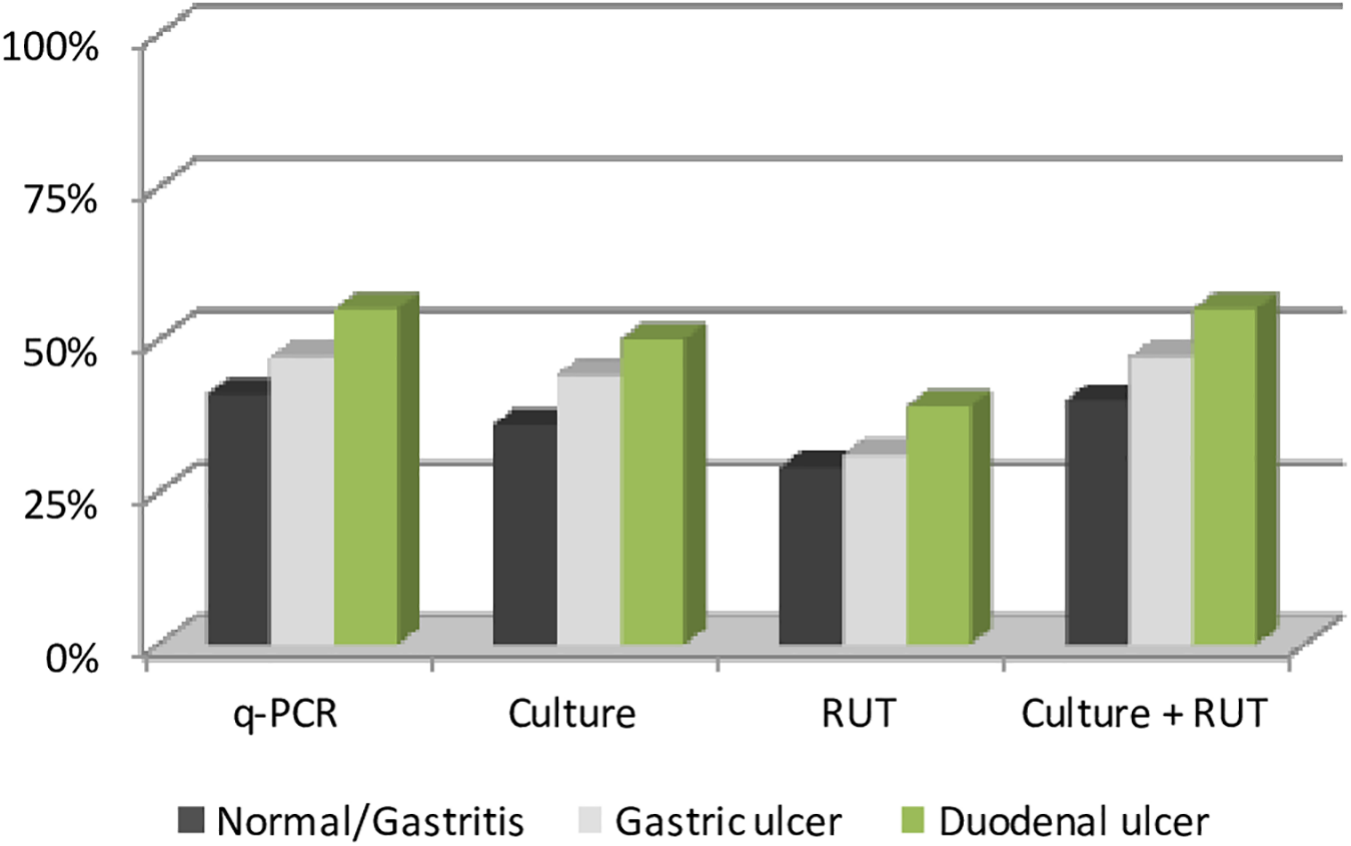
Percentage of biopsies testing positive for *H*. *pylori*.

The percentage of biopsies testing positive for *H*. *pylori* according to diagnostic test used and results of endoscopy are shown graphically in Fig 2.

A total of 196 patients gave results which were concordant using both traditional methods and PCR. Of the non-concordant results, ten biopsies were positive by PCR although showed negative in culture, eight of which were RUT positive. One biopsy was negative by PCR, although positive by both culture and RUT. PCR and culture had the highest agreement (kappa = 0.887), then PCR and RUT (kappa = 0.709) and finally culture and RUT (kappa = 0.645). Table 4 shows concordance between tests.

**Table 4:** Concordance between diagnostic tests.

	Culture		RUT		Culture+RUT	
	Positive	Negative	Positive	Negative	Positive	Negative
*H*. *pylori* positive by PCR	80 (40%)	10 (5%)	63 (32%)	27 (14%)	88 (44%)	2 (1%)
*H*. *pylori* negative by PCR	1 (0.5%)	108 (54%)	1 (0.5%)	108 (54%)	1 (0.5%)	108 (54%)

### 3.3. Q-PCR results and analysis of *H*. *pylori* DNA load

**Fig 3:**
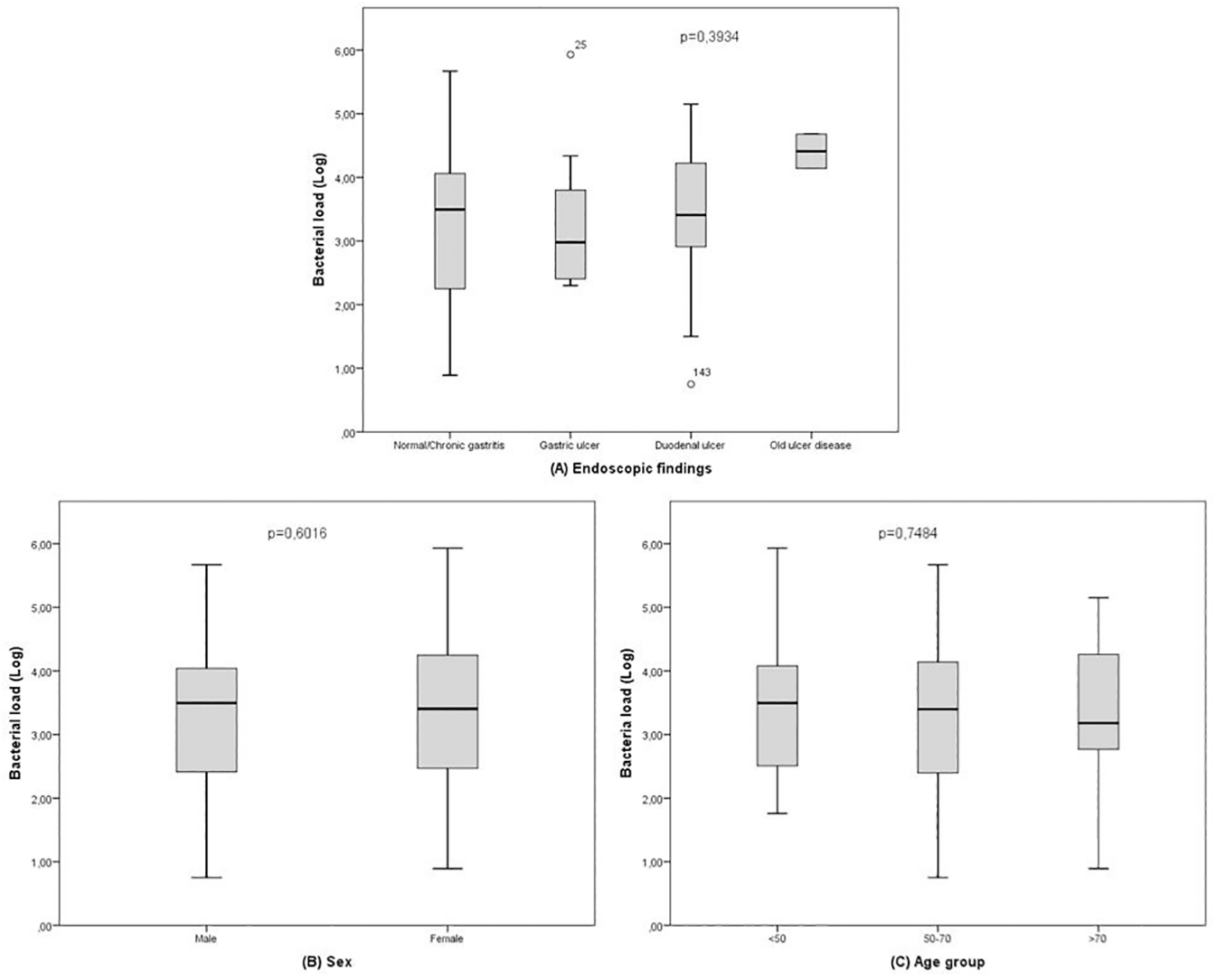
Relationship between average *H*. *pylori* bacterial load and endoscopic findings, sex and age.

The average *H*. *pylori* bacterial load (DNA copies/10^5^ cells) in our patients was 3.34±1.06 log (0.75–5.93). In relation to endoscopic findings, the average bacterial load was 3.22±1.10 log (0.89–5.67) in patients with chronic gastritis/normal endoscopy, 3.29±1.04 log (2.30–5.93) in patients with gastric ulcer, 3.46±1.02 log (0.75–5.15) in patients with duodenal ulcer and 4.41±0.38 log in two patients with healed ulcer (p = ns). Bacterial load according to sex was 3.27±1.04 log (0.75–5.67) in male and 3.39±1.08 log (0.89–5.93) in female (p = ns). By age groups, *H*. *pylori* bacterial load was 3.38±0.99 log (1.76–5.93) in patients under 50 years, 3.29±1.10 log (0.75–5.67) in patients between 50 and 70 years and 3.40±1.13 log (0.89–5.15) in patients over 70 years (p = ns) (Fig 3).

**Fig 4:**
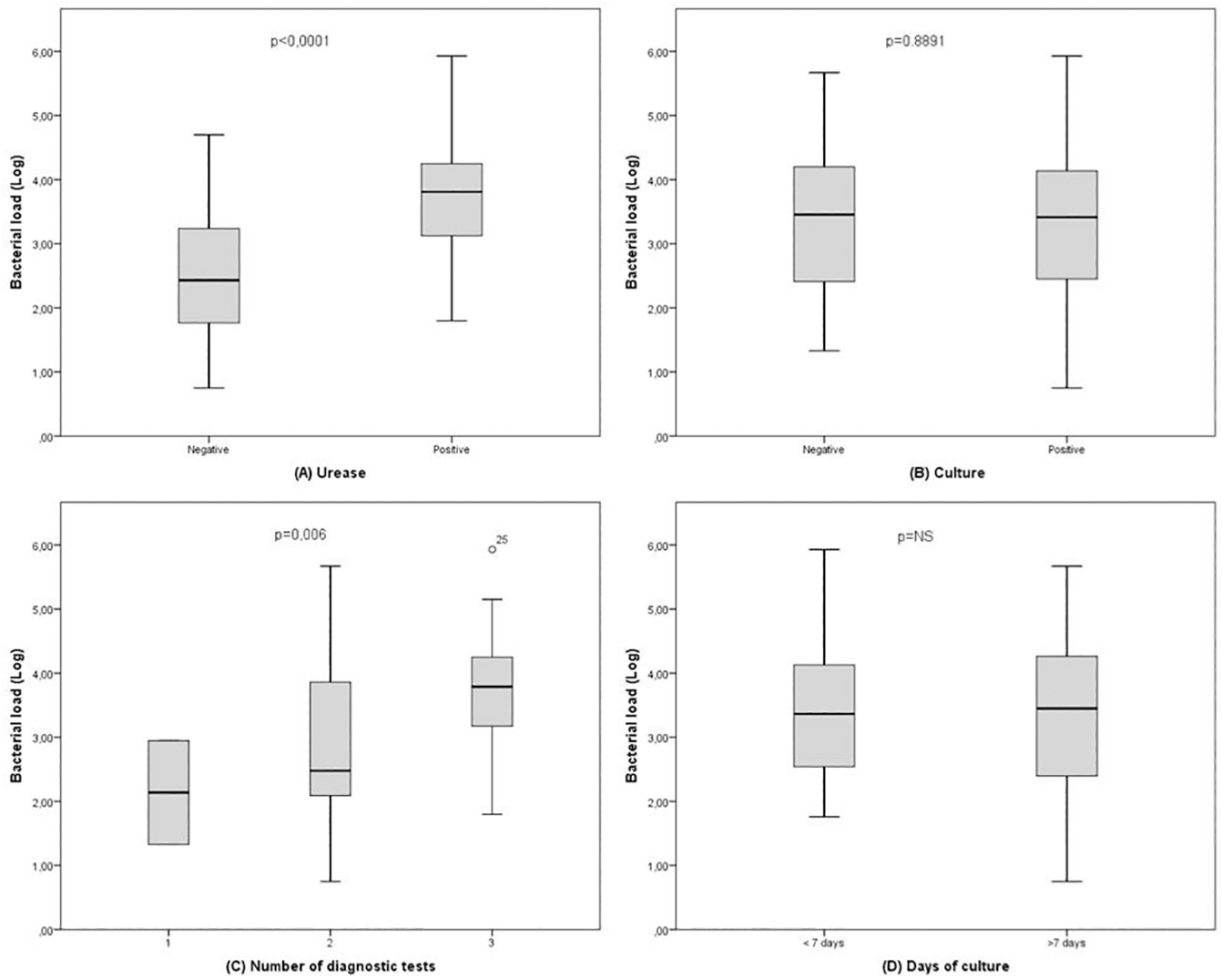
Comparison of average *H*. *pylori* DNA between different diagnostic tests.

With respect to the different diagnostic tests, the average number of copies of *H*. *pylori* DNA in samples testing positive by both RUT and PCR was 3.67±0.89 log (1.80–5.93) and 2.58±1.05 log (0.75–4.70) in PCR positive/RUT negative samples (p<0.0001). In relation to culture results, average bacterial load in those positive both by culture and PCR was 3.34±1.04 log (0.75–5.93), and 3.39±1.28 log (1.33–5.67) in those positive by PCR but negative by culture (p = ns) (Fig 4A and Fig 4B).

Out of 91 positive *H*. *pylori* cases, 2 (2%) were detected by one method alone, 34 (37%) by two methods and 55 (6%) by each of the three methods. The average *H*. *pylori* bacterial load detected in samples found positive by all methods was 3.66±0.85 log (1.80–5.93) and 2.87±1.17 log (0.75–5.67) in samples found positive by two methods (p = 0.006) (Fig 4C).

Although both q-PCR and culture have high detection rates, while PCR gives results quickly, only 38 (42%) patients gave positive culture results within seven days. Speed of culture results however had no bearing on number of copies, average number for positive culture results before 7 days being 3.37±0.93 log (1.76–5.93) and those after 7 days being 3.32±1.15 log (0.75–5.67) (p = ns) (Fig 4D).

## Discussion

Traditional diagnosis of *H*. *pylori* is performed using a combination of invasive and non-invasive diagnostic tests, due to limitations in terms of sensitivity and/or specificity of the various methods used separately [11,12]. Historically, the combination of RUT and culture, both invasive techniques, has been considered the gold standard from the microbiological point of view. Culture has the great advantage of allowing antimicrobial susceptibility to be tested and provides high specificity. However, it is technically difficult, costly and time consuming because the fastidious nature of *H*. *pylori* requires very specific culture conditions and long incubation [7,13–15]. The other traditional invasive microbiological test, RUT, has the advantage of providing results in 24–72 hours, but it has the limitation of requiring at least 10^5^ bacteria to obtain good enough sensitivity and it may not be specific in the presence of other urease-positive bacteria [12,16–19]. To date, none of these techniques allows the number of bacteria per sample to be quantified. Studies based on molecular techniques have been performed to detect *H*. *pylori* DNA in biopsy samples, with high sensitivity and specificity, and some also allow bacterial quantification [8,20–23]. In the present study, a quantitative proprietary real-time PCR method for the diagnosis of *H*. *pylo*ri was designed. This PCR is based on a real time system that allows quantification, shorter diagnosis times, and at a low price. The *H*. *pylori* urease gene amplification designed was able to detect 10 copies, a limit of detection low enough to be very useful in case diagnosis, even with compromised samples.

What is more, this method showed total specificity since it did not detect any of the other colonising bacteria from the same habitat as *H*. *pylori*.

This q-PCR also allows quantification of bacterial load expressed per 10^5^ cells. The use of normalized counts, focusing on sample quality rather than absolute numbers enables comparisons between samples to be made. The possibility of quantifying bacterial load has been proven to be useful in previous studies, where the variability of *H*. *pylori* DNA load for different histopathological parameters, different endoscopic findings or the different results of traditional diagnostic techniques were described [20,21,24,25]. In this study such differences were not found, though it must be acknowledged that sample numbers were low and details of symptoms and endoscopic findings were not available for all patients.

Of the 199 patients included in this study, *H*. *pylori* was found in 91 (46%) when all methods were considered together, a prevalence similar to other studies [25–27]. The sensitivity of the in-house PCR used here was similar to that obtained using two traditional methods together (culture plus RUT). Concordance between the combination of traditional methods and q-PCR was almost 100%. When compared separately with each of these two traditional methods, q-PCR was more sensitive than culture and far more sensitive than RUT. Two patients tested positive by q-PCR but not by traditional methods. Both samples had lower bacterial loads than could not be detected by the other methods. The average number of copies of *H*. *pylori* DNA of these samples was lower than those samples testing positive by two or three techniques. Moreover, the possibility of false positive results was excluded by repeating the test several times and achieving the same *cycle threshold*. One patient was positive by traditional methods and negative by q-PCR, which may be due to the non-uniform distribution of *Helicobacter* in gastric mucosa.

One of the great advantages of q-PCR over traditional methods is the decrease in response time. Indeed, culturing *H*. *pylori* required more than 7 days for results to be reported for most of the patients (80%). RUT could be compared to q-PCR in terms of response time, i.e. giving results within 24–72 hours, but its sensitivity was considerably lower (32%). As said before, RUT sensitivity is greatly influenced by bacterial density, requiring a minimum number of the organisms per biopsy, and varies significantly from one study to another [24,26].

It must be recognised, however, that traditional culture techniques are able to provide information about antimicrobial susceptibility, which is essential in order to establish the appropiate antibiotic treatment, and ensure local resistance data is taken into account in adjusting the empirical treatments. This technique could be improved by also considering with the addition of detection of genomic resistance by searching for specific mutations in the *H*. *pylori* genome. Further studies focused on this issue need to be carried out.

We found differences between bacterial load in samples testing positive in two or three traditional diagnostic tests and those which gave only one positive result. Quantification of microorganisms in the gastric mucosa by q-PCR would complement the limitations of traditional methods making it possible to differentiate between asymptomatic colonisation and infection [21,28,29]. Indeed, a number of dyspeptic patients could be diagnosed with *H*. *pylori* infection, patients who may currently be being misdiagnosed due to the low sensitivity of the traditional assays rather than absence of infection.

No bacterial load difference was associated with clinical symptoms. As said above, further work focused on more specific clinical symptoms and using a greater number of patients could help to clarify this point.

In conclusion, we developed a q-PCR with satisfactory results in terms of sensitivity, specificity, low cost and easy processing, though of course, supplementary studies with a greater number of samples would provide stronger data. The good results achieved in this study, coupled with the low cost and easy processing make this technique useful for both large and small microbiology laboratories, both public and private. It might even be possible to look for a non-invasive technique, such as pharyngeal swabs or stool samples where this technique could be used. Nevertheless, culture still remains an important tool as it provides information in terms of antimicrobial susceptibility which is necessary to choose the right treatment option [6,30]. Molecular assays are available to detect DNA point mutations associated with clarithromycin and metronidazole resistance [31,32] but their high price makes it prohibitive for small laboratories to use them. The technique described here could provide a first step to including the detection of DNA mutations associated with antibiotic resistance.

## References

[1] Hunt RH, Xiao SD, Megraud F, Leon-Barua R, Bazzoli F, van der Merwe S, et al Helicobacter pylori in developing countries. World gastroenterology organisation global guideline. J Gastrointest Liver Dis. 2011;20(3):299–304. 21961099

[2] Malfertheiner P, Megraud F, O’Morain CAC a., Atherton J, Axon ATR, Bazzoli F, et al Management of Helicobacter pylori infection—the Maastricht IV/ Florence Consensus Report. Gut [Internet]. 2012;61(5):646–64. doi: 10.1136/gutjnl-2012-302084 22491499 10.1136/gutjnl-2012-302084

[3] Gasbarrini G, Racco S, Franceschi F, Miele L, Cammarota G, Grieco A, et al [Helicobacter pylori infection: from gastric to systemic disease]. Recenti Prog Med [Internet]. 2010 1;101(1):27–33. Available from: http://www.ncbi.nlm.nih.gov/pubmed/20391683 20391683

[4] McColl KEL. Clinical practice. Helicobacter pylori infection. N Engl J Med. 2010 4;362(17):1597–604. doi: 10.1056/NEJMcp1001110 20427808 10.1056/NEJMcp1001110

[5] Malfertheiner P, Megraud F, O’Morain CA, Gisbert JP, Kuipers EJ, Axon AT, et al Management of *Helicobacter pylori* infection—the Maastricht V/Florence Consensus Report. Gut. 2016 10;gutjnl-2016-312288.

[6] Gisbert JP, Molina-Infante J, Amador J, Bermejo F, Bujanda L, Calvet X, et al [IV Spanish Consensus Conference on Helicobacter pylori infection treatment]. Gastroenterol Hepatol. 2016 6; 10.1016/j.gastrohep.2016.05.003 27342080

[7] Patel SK, Pratap CB, Jain AK, Gulati AK, Nath G. Diagnosis of Helicobacter pylori: What should be the gold standard? [Internet]. Vol. 20, World Journal of Gastroenterology. Baishideng Publishing Group Inc; 2014 [cited 2016 Nov 23]. p. 12847–59. Available from: http://www.ncbi.nlm.nih.gov/pubmed/25278682 doi: 10.3748/wjg.v20.i36.12847 25278682 10.3748/wjg.v20.i36.12847 PMC4177467

[8] Clayton CL, Kleanthous H, Coates PJ, Morgan DD. Detection of Helicobacter pylori by using the polymerase chain reaction. J Clin Microbiol [Internet]. 1992;29(4):192–200. Available from: http://www.ncbi.nlm.nih.gov/pubmed/1890169%5Cnhttp://www.pubmedcentral.nih.gov/articlerender.fcgi?artid=PMC269854 10.1128/jcm.30.1.192-200.1992 PMC265019 1734052

[9] Granstrom M, Lehours P, Bengtsson C, Mégraud F. Diagnosis of Helicobacter pylori. Helicobacter. 2008 10;13 Suppl 1:7–12. 18783515 10.1111/j.1523-5378.2008.00637.x

[10] Alvarez-Argüelles ME, de Ona M, Rojo-Alba S, Torrens-Muns M, Junquera-LLaneza ML, Boga JA, et al Quantification of human papilloma virus (HPV) DNA using the Cobas 4800 system in women with and without pathological alterations attributable to the virus. J Virol Methods. 2015;222:95–102. doi: 10.1016/j.jviromet.2015.05.016 26057221 10.1016/j.jviromet.2015.05.016

[11] Cirak MY, Akyön Y, Mégraud F. Diagnosis of Helicobacter pylori. Helicobacter [Internet]. 2007 10;12 Suppl 1:4–9. Available from: http://www.ncbi.nlm.nih.gov/pubmed/17727453 17727453 10.1111/j.1523-5378.2007.00542.x

[12] Pourakbari B, Ghazi M, Mahmoudi S, Mamishi S, Azhdarkosh H, Najafi M, et al Diagnosis of Helicobacter pylori infection by invasive and noninvasive tests. Braz J Microbiol [Internet]. 2013 [cited 2016 Nov 22];44(3):795–8. Available from: http://www.ncbi.nlm.nih.gov/pubmed/24516421 doi: 10.1590/S1517-83822013005000052 24516421 10.1590/S1517-83822013005000052 PMC3910191

[13] Lai YC, Wang TH, Huang SH, Yang SS, Wu CH, Chen TK, et al Density of Helicobacter pylori may pylori may affect the efficacy of eradication therapy and ulcer healing in patients with active duodenal ulcers. World J Gastroenterol. 2003;9(7):1537–40. doi: 10.3748/wjg.v9.i7.1537 12854158 10.3748/wjg.v9.i7.1537 PMC4615499

[14] Hachem CY, Clarridge JE, Evans DG, Graham DY. Comparison of agar based media for primary isolation of Helicobacter pylori. J Clin Pathol [Internet]. 1995 8 [cited 2016 Nov 22];48(8):714–6. Available from: http://www.ncbi.nlm.nih.gov/pubmed/7560195 7560195 10.1136/jcp.48.8.714 PMC502795

[15] Mégraud F, Bessède E, Lehours P. Diagnosis of Helicobacter pylori Infection. Helicobacter. 2014;19(S1):6–10. 25167939 10.1111/hel.12161

[16] Wang Y-K, Kuo F-C, Liu C-J, Wu M-C, Shih H-Y, Sophie SW Wang, et al Diagnosis of Helicobacter pylori infection: Current options and developments. World J Gastroenterol [Internet]. 2015 10 28 [cited 2016 Nov 22];21(40):11221 Available from: http://www.ncbi.nlm.nih.gov/pubmed/26523098 doi: 10.3748/wjg.v21.i40.11221 26523098 10.3748/wjg.v21.i40.11221 PMC4616200

[17] Graham DY, Opekun AR, Hammoud F, Yamaoka Y, Reddy R, Osato MS, et al Studies regarding the mechanism of false negative urea breath tests with proton pump inhibitors. Am J Gastroenterol [Internet]. 2003 5;98(5):1005–9. Available from: http://www.ncbi.nlm.nih.gov/pubmed/12809820 doi: 10.1111/j.1572-0241.2003.07426.x 12809820 10.1111/j.1572-0241.2003.07426.x

[18] Lee JM, Breslin NP, Fallon C, O’Morain CA. Rapid urease tests lack sensitivity in Helicobacter pylori diagnosis when peptic ulcer disease presents with bleeding. Am J Gastroenterol [Internet]. 2000 5;95(5):1166–70. Available from: http://www.ncbi.nlm.nih.gov/pubmed/10811322 doi: 10.1111/j.1572-0241.2000.02004.x 10811322 10.1111/j.1572-0241.2000.02004.x

[19] Yakoob J, Jafri W, Abbas Z, Abid S, Islam M, Ahmed Z. The Diagnostic Yield of Various Tests for Helicobacter pylori Infection in Patients on Acid-Reducing Drugs. Dig Dis Sci [Internet]. 2008 1 12;53(1):95–100. Available from: http://link.springer.com/10.1007/s10620-007-9828-y doi: 10.1007/s10620-007-9828-y 17497222 10.1007/s10620-007-9828-y

[20] Kobayashi D, Eishi Y, Ohkusa T, Ishige I, Suzuki T, Minami J, et al Gastric mucosal density of Helicobacter pylori estimated by real-time PCR compared with results of urea breath test and histological grading. J Med Microbiol. 2002;51(4):305–11. doi: 10.1099/0022-1317-51-4-305 11926735 10.1099/0022-1317-51-4-305

[21] Ribeiro ML, Ecclissato CC, Mattos RG, Mendonca S, Pedrazzoli J Jr. Quantitative real-time PCR for the clinical detection of Helicobacter pylori. Genet Mol Biol. 2007;30:431–4.

[22] Schabereiter-gurtner C, Hirschl AM, Dragosics B, Hufnagl P, Puz S, Kova Z, et al Novel Real-Time PCR Assay for Detection of Helicobacter pylori Infection and Simultaneous Clarithromycin Susceptibility Testing of Stool and Biopsy Specimens. 2004;42(10):4512–8. doi: 10.1128/JCM.42.10.4512-4518.2004 15472302 10.1128/JCM.42.10.4512-4518.2004 PMC522301

[23] Simala-Grant JL, Taylor DE. Molecular biology methods for the characterization of Helicobacter pylori infections and their diagnosis. Apmis. 2004;112(11–12):886–97. 15688524 10.1111/j.1600-0463.2004.apm11211-1211.x

[24] Chou CH, Sheu BS, Yang HB, Cheng PN, Shin JS, Chen CY, et al Clinical assessment of the bacterial load of Helicobacter pylori on gastric mucosa by a new multi-scaled rapid urease test. J Gastroenterol Hepatol [Internet]. 1997 1;12(1):1–6. Available from: http://www.ncbi.nlm.nih.gov/pubmed/9076614 9076614 10.1111/j.1440-1746.1997.tb00336.x

[25] Shukla SK, Prasad KN, Tripathi A, Ghoshal UC, Krishnani N, Nuzhat H. Quantitation of Helicobacter pylori ureC gene and its comparison with different diagnostic techniques and gastric histopathology. J Microbiol Methods [Internet]. 2011;86(2):231–7. doi: 10.1016/j.mimet.2011.05.012 21624400 10.1016/j.mimet.2011.05.012

[26] Al-Moayad EE, Alghalibi SM, Al-Shamahy HA, Nasher AT, Al-hebshi NN. Normalized real-time PCR for diagnosis of H. pylori infection. Qatar Med J [Internet]. 2014 [cited 2016 Nov 22];2014(2):19 Available from: http://www.ncbi.nlm.nih.gov/pubmed/25745602 10.5339/qmj.2014.19 PMC4344986 25745602

[27] Belda S, Saez J, Santibáñez M, Rodríguez JC, Galiana A, Sola-Vera J, et al Quantification of Helicobacter pylori in gastric mucosa by real-time polymerase chain reaction: comparison with traditional diagnostic methods. Diagn Microbiol Infect Dis. 2012;74(3):248–52. doi: 10.1016/j.diagmicrobio.2012.07.007 22921814 10.1016/j.diagmicrobio.2012.07.007

[28] Blaser MJ. Who are we? Indigenous microbes and the ecology of human diseases. EMBO Rep. 2006;7(10):956–60. doi: 10.1038/sj.embor.7400812 17016449 10.1038/sj.embor.7400812 PMC1618379

[29] Misra V, Misra SP, Singh MK, Singh PA, Dwivedi M. Prevalence of H. pylori in patients with gastric cancer. Indian J Pathol Microbiol [Internet]. 2007 10 [cited 2016 Nov 22];50(4):702–7. Available from: http://www.ncbi.nlm.nih.gov/pubmed/18306532 18306532

[30] Fallone CA, Chiba N, van Zanten SV, Fischbach L, Gisbert JP, Hunt RH, et al The Toronto Consensus for the Treatment of Helicobacter pylori Infection in Adults. Gastroenterology [Internet]. 2016 7 [cited 2016 Nov 22];151(1):51–69.e14. Available from: http://linkinghub.elsevier.com/retrieve/pii/S0016508516301081 doi: 10.1053/j.gastro.2016.04.006 27102658 10.1053/j.gastro.2016.04.006

[31] Agudo S, Alarcón T, Urruzuno P, Martínez MJ, López-Brea M. Detection of Helicobacter pylori and clarithromycin resistance in gastric biopsies of pediatric patients by using a commercially available real-time polymerase chain reaction after NucliSens semiautomated DNA extraction. Diagn Microbiol Infect Dis. 2010;67(3):213–9. doi: 10.1016/j.diagmicrobio.2010.02.021 20542201 10.1016/j.diagmicrobio.2010.02.021

[32] Cambau E, Allerheiligen V, Coulon C, Corbel C, Lascols C, Deforges L, et al Evaluation of a new test, GenoType HelicoDR, for molecular detection of antibiotic resistance in Helicobacter pylori. J Clin Microbiol [Internet]. 2009 11 [cited 2016 Nov 22];47(11):3600–7. Available from: http://www.ncbi.nlm.nih.gov/pubmed/19759218 doi: 10.1128/JCM.00744-09 19759218 10.1128/JCM.00744-09 PMC2772597

